# PRX5 as a critical driver of colorectal cancer stemness and tumorigenicity

**DOI:** 10.1080/13510002.2026.2673661

**Published:** 2026-05-16

**Authors:** Sung Woo Lee, Sun-Ji Park, Ga Eun Lee, Su-Min Jung, Jaeyeon Lee, San Kwon, Eunbyul Yeom, Dong-Seok Lee, Eui-Hwan Choi

**Affiliations:** a BK21 FOUR KNU Creative BioResearch Group, School of Life Sciences, Kyungpook National University, Daegu, Republic of Korea; b School of Life Sciences & Biotechnology, College of Natural Sciences, Kyungpook National University, Daegu, Republic of Korea; c Department of Physiology, Jeonbuk National University Medical School, Jeonju, Republic of Korea; d School of Life Sciences, College of Natural Sciences, KNU-G LAMP Project Group, KNU-Institute of Basic Sciences, Kyungpook National University, Daegu, Republic of Korea; e Department of Biotechnology, Korea National University of Transportation, Chungbuk, Republic of Korea

**Keywords:** Colorectal cancer, cancer stem cell, Peroxiredoxin 5, ROS, stemness

## Abstract

**Background:**

Cancer stem cells (CSCs) support colorectal cancer progression and therapy resistance, yet the redox regulators that sustain CSC identity are incompletely defined. We investigated the role of peroxiredoxin 5 (PRX5) in CSC formation and tumorigenicity using HCT116 colorectal cancer models.

**Methods:**

CSCs were enriched by serum-free spheroid culture and characterized by qPCR and western blotting for pluripotency and surface markers. PRX5 expression was regulated via siRNA or shRNA gene disruption and overexpression. Intracellular ROS was measured with DCF-DA staining. STAT3 activation was analyzed by *p*-STAT3 immunoblot. Functional assays included sphere formation, extreme limiting dilution analysis (ELDA), colony formation, and *in vivo* xenograft tumorigenicity in BALB/c-nu mice.

**Results:**

Spheroid induction selectively upregulated PRX5 among PRX isoforms. Efficient PRX5 knockdown (>95%) reduced OCT4, SOX2, NANOG, and CD133 expression, increased intracellular ROS, lowered *p*-STAT3 levels, and decreased sphere-forming frequency. Conversely, PRX5 overexpression enhanced pluripotency marker expression and proliferation. *In vivo*, PRX5-overexpressing xenografts grew faster, achieving a 2.5-fold greater tumor volume by day 19 and a 1.82-fold higher mean tumor weight compared with controls. Tumor tissues showed elevated OCT4, SOX2, NANOG, CD133, and EPCAM.

**Conclusion:**

PRX5 contributes to maintaining a redox environment associated with STAT3 activation and core stemness programs in CRC, promoting CSC phenotypes and tumorigenicity. Targeting PRX5-mediated redox signaling may warrant further investigation as a potential approach to disrupt CSC maintenance and overcome chemoresistance.

## 1. Introduction

Colorectal cancer (CRC) is a global health challenge and one of the leading causes of cancer-related mortality worldwide [1]. While advancements in early detection and multimodal therapies (including surgical resection, chemotherapy, and targeted agents) have improved initial patient response, high rates of tumor recurrence, distant metastasis, and intrinsic therapeutic resistance often lead to treatment failure [2,3]. This disturbing clinical outcome is attributed to the persistence of a small, highly aggressive subpopulation of CSCs [4]. The latter’s remarkable capacity for self-renewal, differentiation into the heterogeneous tumor mass, and extraordinary tumor-initiating potential enables them to drive tumor progression and therapeutic evasion [4,5]. The human CRC cell line HCT116 is a well-established *in vitro* model for studying the intricate molecular mechanisms behind the generation and proliferation of this crucial CSC phenotype [6,7].

The core identity of CSCs is transcriptionally maintained by a highly regulated network of pluripotency factors, OCT4, SOX2, NANOG, and c-MYC [8,9]. The synergistic and amplified expression of these transcription factors causes colorectal cells to degenerate into a primitive, undifferentiated, and aggressive stem-like state, thereby conferring the key biological hallmarks of cancer stemness. Furthermore, the functional CSC phenotype can be identified by the expression of specific cell surface markers, such as EPCAM, CD24, CD44, and CD133, which can be used to realize the physical isolation and prognostic exploitation of the former [10–12]. The co-expression and dynamic regulation of these cancer stemness transcription factors and surface markers indicate inducement of the CSC state, a process that is required to navigate and survive challenging microenvironments.

CSCs need to maintain viability and functionality during the heightened metabolic activity and resulting oxidative stress found in the tumor microenvironment [13]. CSC cells can upregulate robust antioxidant defense systems to reduce the effect of oxidative stress and preserve the delicate balance required for self-renewal [14,15]. A recent study has further highlighted that CSCs rely on coordinated antioxidant programs and redox-sensitive signaling networks to sustain self-renewal, phenotypic plasticity, therapeutic resistance, and tumor-initiating potential under oxidative stress [16]. Central to this defense is the peroxiredoxin (PRX) family of enzymes, a group of highly conserved thiol-specific peroxidases comprising six distinct isoforms (PRX1-6) [14,17]. These enzymes function across various cellular compartments to regulate cellular redox homeostasis by effectively scavenging and reducing peroxides. Notably, CSCs require a specific low-to-moderate level of reactive oxygen species (ROS) for optimal self-renewal and proliferation since excessive ROS levels can trigger differentiation or apoptosis [18–20]. Therefore, the regulation of this fine-tuned ROS balance is paramount for CSC survival, with PRX5 (primarily localized in the mitochondria and peroxisomes) being particularly important [21–24]. Given its crucial role in regulating ROS within key organelles involved in metabolic reprogramming, we hypothesized that PRX5 could provide a specific, non-redundant role in facilitating the survival and maintenance of the malignant stem-like state.

Despite the known role of antioxidant systems in cancer progression, the specific regulatory function of individual PRX isoforms, particularly PRX5, in the context of CRC stemness and tumorigenicity remains largely undetermined. Therefore, this study was designed to find the functional and mechanistic roles of PRX5 in governing the CSC phenotype in HCT116 cells. We first employed a well-validated spheroid culture method to successfully induce and enrich a CSC cell population and validated the model through the transcriptional and proteomic upregulation of core stemness factors and surface markers. Next, through a comparative analysis of the expression of PRX family members, we identified PRX5 as the predominantly upregulated isoform during CSC induction. Critically, we used both short hairpin (shRNA)-mediated knockout and stable overexpression of PRX5 to uncover the causal link between PRX5 and the malignant CSC phenotype and assess its impact on core stemness marker expression and proliferative capacity. Finally, we provided translational validation by utilizing an in vivo murine xenograft model to directly evaluate the effect of PRX5 overexpression on tumor growth, thereby establishing PRX5 as a critical driver of CRC progression. Collectively, this work provides evidence of the morphological changes, genetic and protein expression, and in vivo functionality of PRX5 to uncover its role as a regulatory component that sustains the transcriptional program underpinning CSC identity and drives tumorigenicity in CRC.

## 2. Methods

### 2.1. Reagents

Recombinant human epidermal growth factor (EGF) and recombinant human basic fibroblast growth factor (bFGF) were obtained from PeproTech (Cranbury, NJ, U.S.A.). B-27 supplement was purchased from Thermo Fisher Scientific (Waltham, MA, U.S.A). Insulin, *N*-acetyl-L-cysteine (NAC), and STAT3 Inhibitor III, WP1066 (WP1066), were purchased from Sigma–Aldrich (St. Louis, MO, U.S.A.). Detailed information regarding the reagents used in this study, including supplier names and catalog numbers, is provided in Table S2.

### 2.2. Cell culture and spheroid formation

HCT116 human colon cancer cells were purchased from the American Type Culture Collection (Manassas, VA, U.S.A.). The cells were maintained in Dulbecco’s Modified Eagle’s medium (DMEM) (Welgene, Daegu, Korea) supplemented with 10% fetal bovine serum (FBS; Thermo Fisher Scientific), 100 U/mL penicillin, and 100 μg/mL streptomycin (Welgene) at 37 °C in a humidified incubator containing 5% CO_2_. For spheroid formation, HCT116 cells were detached with 1 x Trypsin-EDTA (Welgene) to prepare a single-cell suspension, which was seeded into ultra-low attachment 96-well plates at a density of 1 × 10^3^ cells/well. The spheroids were maintained for up to 12 days in DMEM/F-12 medium supplemented with 20 ng/mL EGF, 10 ng/mL bFGF, 5 μg/mL insulin, and 1x B-27 supplement (Thermo Fisher Scientific). The culture medium was carefully replaced every 3 days to minimize disturbance to spheroid integrity. For functional modulation experiments, cells were treated with the NAC (1 mM) or WP1066 (10 μM). Treatments were initiated at the time of seeding and reapplied at each medium change. Spheroid formation was monitored using a Cytation 5 Cell Imaging Multimode Reader (Agilent Technologies, Santa Clara, CA, U.S.A.).

### 2.3. Plasmid construction and lentivirus generation

The PRX5 gene was amplified through PCR using the PrimeSTAR® GXL DNA polymerase (Takara Bio Inc., Shiga, Japan). The amplified PRX5 fragments were inserted into pLenti6.3/V5-DEST using EcoRV restriction sites and an In-Fusion® Snap Assembly cloning kit (Takara Bio Inc.), according to the manufacturer’s instructions, to generate a C-terminal V5-tagged construct (pLenti6.3-PRX5). HEK293FT cells were cultured until 70%–80% confluence and transfected with pLenti6.3-PRX5, the packaging vector psPAX2, and enveloping vector pMD.2G using the Effectene transfection reagent (Qiagen, Hilden, Germany). After 12 h, the medium was replaced with fresh complete medium. Lentivirus-containing supernatants were collected 24–48 h later and filtered through a 0.45-μm Minisart syringe filter (Sartorius, Gottingen, Germany). To achieve a high viral titer, the lentivirus-containing medium was concentrated and purified using an Amicon® Ultra-15 centrifugal filter unit (Millipore, Billerica, MA, U.S.A.).

### 2.4. Stable cell lines

To generate stable PRX5-expressing HCT116 cells (HCT116-PRX5) cell line and PRX5-knockdown HCT116 cells (HCT116-PRX5 shRNA), the respective constructed lentivirus was used to infect HCT116 cells in the presence of 8 μg/mL polybrene. After 48 h of incubation, HCT116-PRX5 cells were selected using 8 μg/mL blasticidin (Sigma–Aldrich) for 1 week, whereas HCT116-PRX5 shRNA cells were selected using 1 μg/mL puromycin (Sigma–Aldrich) for 1 week.

### 2.5. RNA interference

siRNA-mediated knockdown was performed using a predesigned AccuTarget™ siRNA (Bioneer, Daejeon, Korea) specific for PRX5. To this end, HCT116 cells were transfected with siPRX5 or a negative control siRNA (siCtrl) using Lipofectamine™ RNAiMAX (Thermo Fisher Scientific) according to the manufacturer’s protocol. The target sequence for PRX5 siRNA was 5′-CAUCUUUGGGAAUCGACGU-3′.

### 2.6. Western blot analysis

Total protein was extracted from cell and tumor tissue lysates using PRO-PREP Protein Extraction Solution (iNtRON Biotechnology, Seongnam, Korea). For tumor tissue samples, lysates were further supplemented with 1× Halt Protease and Phosphatase Inhibitor Cocktail (Thermo Fisher Scientific). The protein lysates were cleared by centrifugation at 16,000 × g and quantified using the Bradford assay (Bio-Rad, CA, U.S.A.) with a Synergy H1 plate reader (Agilent Technologies). Samples containing equal amounts of protein (10–30 μg) were separated by using sodium dodecyl sulfate (SDS)-polyacrylamide gel electrophoresis (PAGE) on 8%–15% SDS gels and transferred onto nitrocellulose membranes (Pall Corporation, Pensacola, FL, U.S.A.). The membranes were then blocked with 5% skimmed milk (BD Biosciences, CA, U.S.A.) and incubated overnight at 4 °C with the following primary antibodies: GAPDH (Santa Cruz Biotech., 1:20,000), OCT4 (Cell Signaling Technology; CST, 1:1,000), NANOG (CST, 1:1,000), SOX2 (CST, 1:1,000), EPCAM (CST, 1:1,000), CD133 (CST, 1:1000), PRX1 (AbFrontier, 1:1,000), PRX2 (ProteinTech, 1:1,1000), PRX3 (AbFrontier, 1:1,000), PRX4 (AbFrontier, 1:1,000), PRX5 (AbFrontier, 1:1,000), PRX6 (AbFrontier, 1:1,000), and V-5 tag (Enogene, 1:1,000). The membranes were washed five times with 10 mM Tris–HCl (pH 7.5) containing 150 mM NaCl and 0.1% Tween-20 (TBST) and then incubated for 1 hour at room temperature with horseradish peroxidase-conjugated goat anti-rabbit and anti-mouse secondary antibodies (Thermo Fisher Scientific). Subsequently, the membranes were washed six times with TBST to remove non-specifically bound secondary antibodies. Immunoreactive bands were detected using NICSRO-WEST ECL PICO (BIONICS, Seoul, Korea) according to the manufacturer's instructions, and band intensities were analyzed using the ChemiDoc MP Imaging System (Bio-Rad, Hercules, CA, U.S.A.). The specifications of all antibodies, such as their suppliers and catalog numbers, are summarized in Table S2.

### 2.7. Quantitative PCR (qPCR)

Total RNA was extracted from tumor tissues and cells using a Ribospin™ II kit (GeneAll, Seoul, Korea). RNA was converted to cDNA using Rocketscript™ Reverse Transcriptase (Bioneer). PCR reactions were conducted in a 96-well plate (Thermo Fisher Scientific) using the TB green master mix (Takara Bio Inc.). The list of primers used is provided in Table S1.

### 2.8. Colony formation assay

For colony formation assays, HCT116 and HCT116-PRX5 cells were seeded into 6-well plates at a density of 5 × 10² cells per well in DMEM supplemented with 10% FBS. After incubation for 12 days, the cells were washed with phosphate-buffered saline (PBS) and then fixed and stained simultaneously with a mixed solution containing 6% glutaraldehyde and 0.5% crystal violet (Sigma-Aldrich) for 30 minutes. Images were acquired and analyzed using a Cytation 5 Cell Imaging Multimode Reader (Agilent Technologies) equipped with Gen5 software, which automatically quantified colonies larger than 300 µm in diameter.

### 2.9. Animal care and xenografting

We purchased and used 5-week-old male nude (BALB/c-nu) mice (JA Bio, Suwon, Korea). The mice were housed at 23 °C in a 12:12-hour light/dark cycle. All animal experiments were performed according to national ethical guidelines after approval by the Institutional Animal Care and Use Committee (IACUC) at Kyungpook National University. A bilateral flank xenograft model was established after the mice had reached 6 weeks of age. To this end, HCT116 and HCT116-PRX5 cells (5 × 10⁶ cells in 100 μL) were resuspended in Matrigel (354248, Corning, U.S.A.) diluted 1:2 in cold phosphate-buffered saline and subcutaneously injected into the left and right flanks, respectively, using a 31-gauge syringe (*n* = 5). Cell preparation and injection were carried out in a blinded fashion by different investigators. Randomization was not applied at the animal level because each mouse served as its own control in a paired left–right flank design. At day 4, experiments were continued only in mice in which tumors on both sides had been successfully established (*n* = 4). The tumor size and body were measured every 3 days, and the tumor volume was calculated using the following formula:

$$\mathrm{Tumor}\;\mathrm{volume}\;({mm}^{3})={\left(\mathrm{width}\;(mm)\right)}^{2}\times \mathrm{length}\;(mm)/2$$

In accordance with the approved animal protocol, the tumor burden did not exceed 5% of the host animal’s body weight, and the experiment was terminated on day 19. After termination, the mice were euthanized by a trained researcher via cervical dislocation under 2,2,2-tribromoethanol (Avertin; Sigma-Aldrich), and the tumor masses were then harvested and analyzed.

### 2.10. Immunohistochemistry

Tumor tissues collected from each animal group were fixed in 4% paraformaldehyde, embedded using a Tissue-Tek O.C.T. compound (Sakura Finetek, Torrance, CA, U.S.A.), cut into 12 μm-thick sections, and subjected to immunohistochemistry. Briefly, tissue sections were first incubated with a blocking solution (normal goat serum (10%); Thermo Fisher Scientific) followed by incubation with anti-CD133, and anti-PRX5 antibodies at 4 °C. Subsequently, the tissue sections were labeled using Alexa Fluor 488-conjugated and Alexa Fluor 647-conjugated secondary antibodies (Molecular Probes, OR, U.S.A.) for 1 h and observed under an LSM-800 confocal microscope (Carl Zeiss, Jena, Germany).

### 2.11. Extreme limiting dilution assay (ELDA)

The extreme dilution assay (ELDA) was performed based on a previous publication [25]. HCT116 cells were seeded into ultra-low attachment U-bottom 96-well plates at different doses (1000, 100, 10, and 1), and spheroid formation was measured under a microscope. Spheroid formation results were calculated using the Extreme Limiting Dilution Analysis software [26].

### 2.12. Spheroid imaging, ROS measurement, and morphometric analysis

Spheroids were maintained in ultra-low attachment U-bottom 96-well plates for 12 days (*n* = 10). For intracellular reactive oxygen species (ROS) measurement and morphometric analysis, spheroids were incubated with 5 μM CM-H_2_DCFDA (Thermo Fisher Scientific) and 1 μg/ml Hoechst 33242 (Thermo Fisher Scientific) for 30 min at 37 °C, followed by three washes with PBS. The stained spheroids were imaged by z-stack acquisition using an Operetta CLS high-content imaging system (PerkinElmer, Waltham, MA, U.S.A.), including bright-field imaging. The acquired z-stack images were processed and merged into a single projection image using Harmony software (PerkinElmer), and the mean fluorescence intensity was quantified for analysis. In addition, spheroid circularity was analyzed using ImageJ software (National Institutes of Health, Bethesda, MD, U.S.A.), and circularity was calculated using the formula Circularity = 4π × (Area/Perimeter^2^).

### 2.13. Statistical analysis

The data are presented as the mean ± standard error of the mean (SEM) of three or more independent experiments. Statistically significant differences were determined by using unpaired two-tailed Student’s t-tests, one-way analysis of variance (ANOVA), or two-way ANOVA using the GraphPad Prism 8 software (GraphPad Software, San Diego, CA, U.S.A.). After one-way or two-way ANOVA, Tukey’s post hoc tests were conducted for intergroup comparisons. The results with *p* < 0.05 were considered statistically significant. Asterisks indicate calculated *p*-values as follows: *: *p* < 0.05; **: *p* < 0.01; ***: *p* < 0.001.

## 3. Results

### 3.1. Induction and characterization of a CSC subpopulation of HCT116 cells

**Figure 1.**
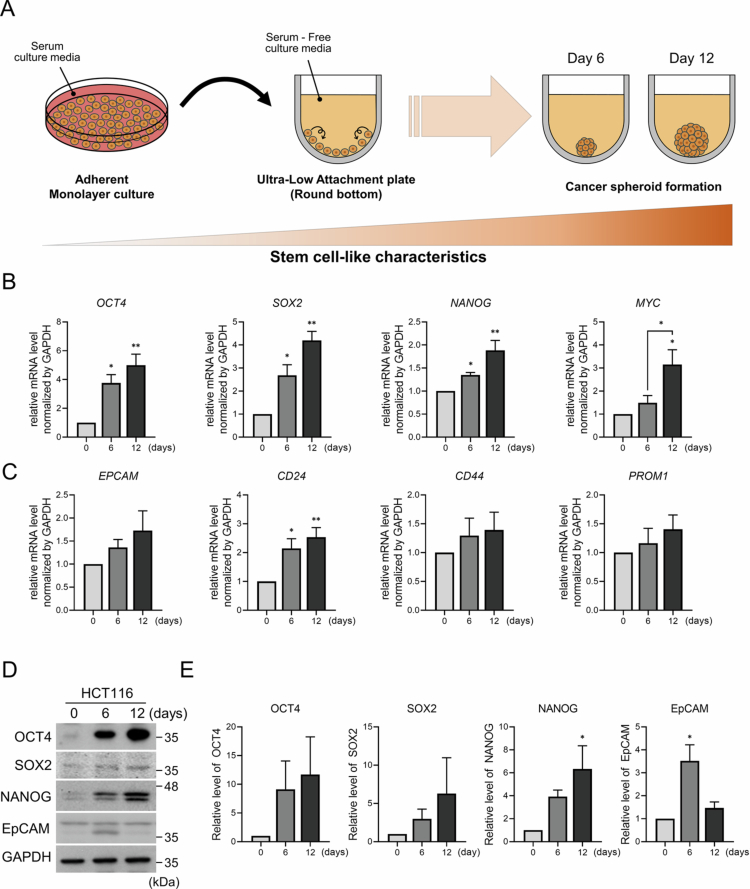
Expression of colon cancer stem cell markers during serum-free spheroid formation of HCT116 cells. (A) The schematic illustrates the procedure for spheroid generation and subsequent analysis. Single-cell suspensions of HCT116 cells were seeded into ultra-low attachment plates and cultured under serum-free conditions for 6–12 days. (B) Relative mRNA expression levels of stemness-related markers (*OCT4, SOX2, NANOG,* and *C-MYC*) and (C) colon cancer stem cell-related markers (*EPCAM, CD24, CD44,* and *CD133*) were determined via qPCR using spheroid samples collected at the indicated time points. (D) Western blot analysis of OCT4, SOX2, NANOG, and EPCAM in spheroid samples obtained from independent cultures under identical serum-free conditions. (E) Densitometric quantification of the protein levels shown in (D). Data are presented as the mean ± standard error of the mean (SEM) of at least three independent experiments. Statistical significance was determined using one-way analysis of variance (ANOVA) with Tukey’s post hoc tests (*: *p* < 0.05; **: *p* < 0.01; ***: *p* < 0.001).

To investigate the acquisition of CSC-like properties in HCT116 CRC cells, we employed a well-established spheroid-based culture method. This approach selectively enriches for cells with stem-like characteristics by exploiting their ability to survive and proliferate under adhesion-independent, serum-free conditions. As illustrated in the experimental scheme in Figure 1A, parent HCT116 cells were cultured in a specialized serum-free medium supplemented with EGF and bFGF, which are critical for the maintenance and proliferation of CSCs. Upon employing the specialized culture conditions, we observed the formation of non-adherent 3D CSC aggregates (termed spheroids) within the HCT116 cell population (Figure S1). This selective enrichment resulted in a distinct subpopulation capable of sustained proliferation without substrate attachment, a morphological feature distinct from the typical adherent growth of HCT116 cells.

To validate the cancer stemness of the enriched spheroid-forming cells, we first performed qPCR to measure the mRNA expression of the core pluripotency transcription factors OCT4, SOX2, NANOG, and C-MYC. These genes are master regulators of pluripotency that are known to be upregulated in CSCs, thereby maintaining their self-renewal capability. The expression levels of all four genes were significantly upregulated in the 12-day spheroid cultures compared to the adherent parental cells (day 0) more than 5-fold for OCT4, 4.1-fold for SOX2, 1.7-fold for NANOG, and 3.1-fold for C-MYC (Figure 1B). This significant increase provides molecular evidence that profound transcriptional reprogramming toward a primitive, undifferentiated state was induced in the spheroid culture.

Further validation of the enriched CSC population was performed by quantifying the mRNA expression of four well-known CSC surface markers commonly used to identify and isolate CSCs across various cancer types: EPCAM, CD24, CD44, and CD133. After 12 days of culturing the spheroids, these markers were significantly upregulated, with their mRNA levels increasing by 1.8-fold for EPCAM, 2.3-fold for CD24, 1.4-fold for CD44, and 1.4-fold for CD133 (Figure 1C). Note that the increases in the expression of these surface markers are more modest than those for the core transcription factors (Figure 1C). This difference is likely due to the distinct roles of these gene sets: while OCT4, SOX2, NANOG, and C-MYC orchestrate the core stemness program at the genetic level, the expression of surface markers is a downstream consequence of this reprogramming and reflects changes in cell-to-cell interactions and functional properties that can delay manifestation.

Nonetheless, the upregulation of the CD24, CD44, and CD133 markers associated with tumorigenicity and therapy resistance provides important evidence that a significant CSC subpopulation was successfully enriched. Furthermore, we confirmed that the cancer stemness characteristics observed at the mRNA level were consistent at the protein level. The protein expression levels of OCT4, SOX2, NANOG, and EPCAM were notably higher in HCT116 cells after 6 and 12 days of CSC induction compared to day 0 (Figure 1D and E). The correlation between the upregulation of mRNA and protein expression levels validates that the transcriptional level changes were successfully translated into functional proteins, thereby supporting our findings. Together, these results provide evidence that our spheroid culture method effectively induced a CSC subpopulation of HCT116 cells, which was validated through the analysis of core stemness factors and CSC surface marker expression.

### 3.2. PRX5 is a key upregulated antioxidant in HCT116 CSCs

**Figure 2.**
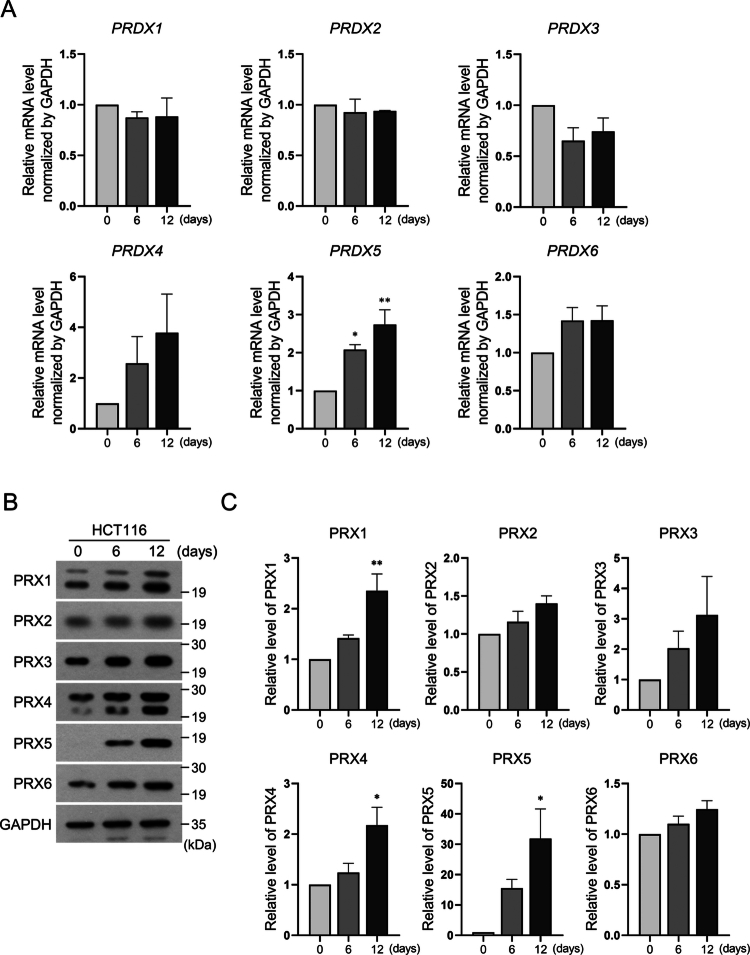
PRX family expression dynamics during spheroid formation of HCT116 cells. (A) Relative mRNA expression levels of *PRX 5* were determined by using qPCR using spheroid samples collected at the indicated time points (0, 6, and 12 days). (B) Western blot analysis of PRX isoforms (PRX1-6) in HCT116 spheroids collected at the indicated time points, showing a marked relative increase in PRX5 protein levels during spheroid development. (C) Quantification of PRX protein levels shown in (B). Data are presented as the mean ± SEM from at least three independent experiments. Statistical significance was determined using one-way ANOVA followed by Tukey’s post hoc tests (*: *p* < 0.05; **: *p* < 0.01; ***: *p* < 0.001).

To determine the molecular mechanisms underlying the acquisition of CSC-like properties, we focused on the expression of the PRX family of antioxidant proteins, which are known to be critical for maintaining cellular redox homeostasis. We started by examining the mRNA expression level of PRX5 in HCT116 cells during their differentiation into the CSC-like state, which was upregulated by approximately 1.7-fold over the 12-day induction period compared to day 0 (Figure 2A). This finding suggests a potential link between PRX5 and the CSC phenotype.

Next, we extended our investigation to the protein expression levels of the entire PRX family. While the expression levels of PRX1-6 all showed slight increases during the CSC differentiation process, the upregulation of PRX5 was notably more pronounced (Figure 2B). This differential expression pattern highlights a preferential upregulation of PRX5, suggesting a potential role in HCT116 CSCs (Figure 2B and C). Unlike other PRX family members that are more broadly expressed in various cellular compartments, PRX5 showed the most prominent induction during spheroid formation, suggesting a potential association with redox regulation in the CSC-like phenotype of HCT116 cells.

### 3.3. PRX5 inhibition suppresses cancer stemness markers and the CSC phenotype in HCT116 cells

**Figure 3.**
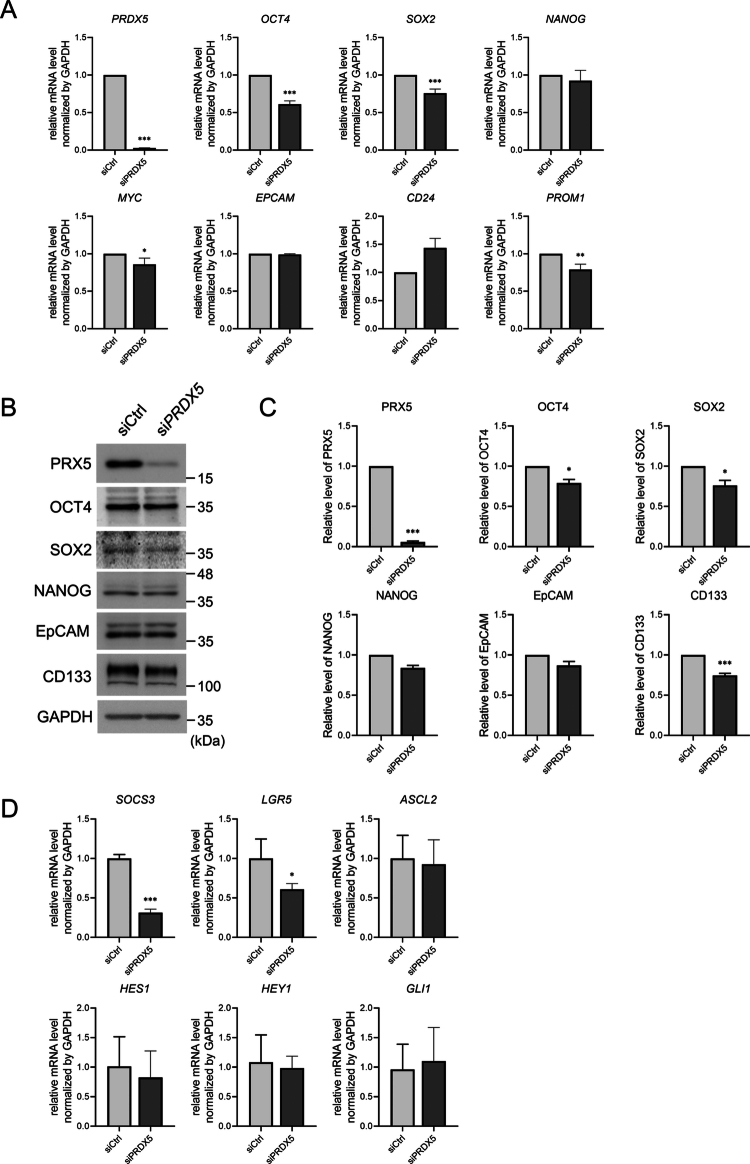
PRX5 knockdown decreases stemness-associated gene and protein expression in HCT116 spheroids. PRX5 knockdown decreases stemness-associated gene and protein expression in HCT116 spheroids. (A) Relative mRNA expression levels of PRX5, OCT4, SOX2, NANOG, C-MYC, CD24, CD133, and EPCAM were determined by using qPCR on day-12 spheroids derived from siCtrl- and siPRX5-transfected HCT116 cells. (B) Western blot analysis of PRX5 and the cancer stemness-related proteins CD133, OCT4, SOX2, NANOG, and EPCAM in siCtrl and PRX5-knockdown spheroids, showing decreased protein expression following PRX5 depletion. (C) Quantification of the protein levels shown in (B). (D) Relative mRNA expression levels of *SOCS3*, *LGR5*, *ASCL2*, *HES1*, *HEY1*, and *GLI1* determined by using qPCR. Data are presented as the mean ± SEM from at least three independent experiments. Statistical significance was determined using an unpaired two-tailed t-test (*: *p* < 0.05; **: *p* < 0.01; ***: *p* < 0.001).

We first established HCT116 CSCs via a 12-day differentiation induction protocol, which was confirmed by the successful upregulation of the core pluripotency factors OCT4, SOX2, NANOG, and c-MYC (Figure 1B). Subsequent quantitative analysis of PRX family members in induced CSCs revealed a significantly larger increase in PRX5 expression compared with that of the other PRX isoforms (Figure 2C), thereby suggesting a specific role for PRX5 in maintenance of the CSC state. To characterize this role, we utilized an siRNA targeting PRX5 (si*PRX5*) to efficient silencing with over 97% suppression of PRX5 mRNA expression (Figure 3A). The PRX5 knockdown resulted in an impaired of the stemness phenotype, as evidenced by the global downregulation of the core stemness transcription factors OCT4, SOX2, NANOG, and c-MYC (Figure 3A). Importantly, the expression levels of OCT4, SOX2, and c-MYC were significantly reduced, demonstrating that PRX5 is essential for maintaining the transcriptional network that supports cancer stemness. Moreover, this functional link was corroborated by the analysis of the CSC surface marker CD133, the expression of which was inhibited by over 23% by si*PRX5* (Figure 3A). Thus, the synchronous suppression of OCT4, SOX2, and CD133 upon PRX5 depletion suggests that PRX5 may function as a regulatory component of the aggressive stem-like properties of HCT116 CSCs.

Western blot analysis confirmed a si*PRX* knockdown efficiency (96%) in HCT116 cells (Figure 3B). In qPCR results, PRX5 silencing significantly attenuated the protein levels of OCT4, SOX2, and NANOG, as well as the CSC marker CD133 (Figure 3C). To examine how PRX5 depletion impacts stemness-related signaling pathways, we quantified the expression of representative downstream target genes by qPCR (Figure 3D). PRX5 knockdown led to a reduction in *SOCS3* expression, indicating diminished STAT3 activity. *LGR5* and *ASCL2*, which are associated with Wnt/β-catenin signaling, also showed a downward trend. In contrast, Notch pathway markers *HES1* and *HEY1* were not induced and did not correlate with PRX5 depletion, while *GLI1* expression remained largely unchanged. These observations indicate that the effects of PRX5 loss are primarily associated with attenuation of STAT3 signaling and, to a lesser extent, Wnt-related transcriptional programs. Together, these results indicate that PRX5 is required for maintaining the CSC phenotype and its associated transcriptional and signaling networks in HCT116 cells.

### 3.4. PRX5 overexpression potentiates stemness features and proliferative capacity in HCT116 cells

**Figure 4.**
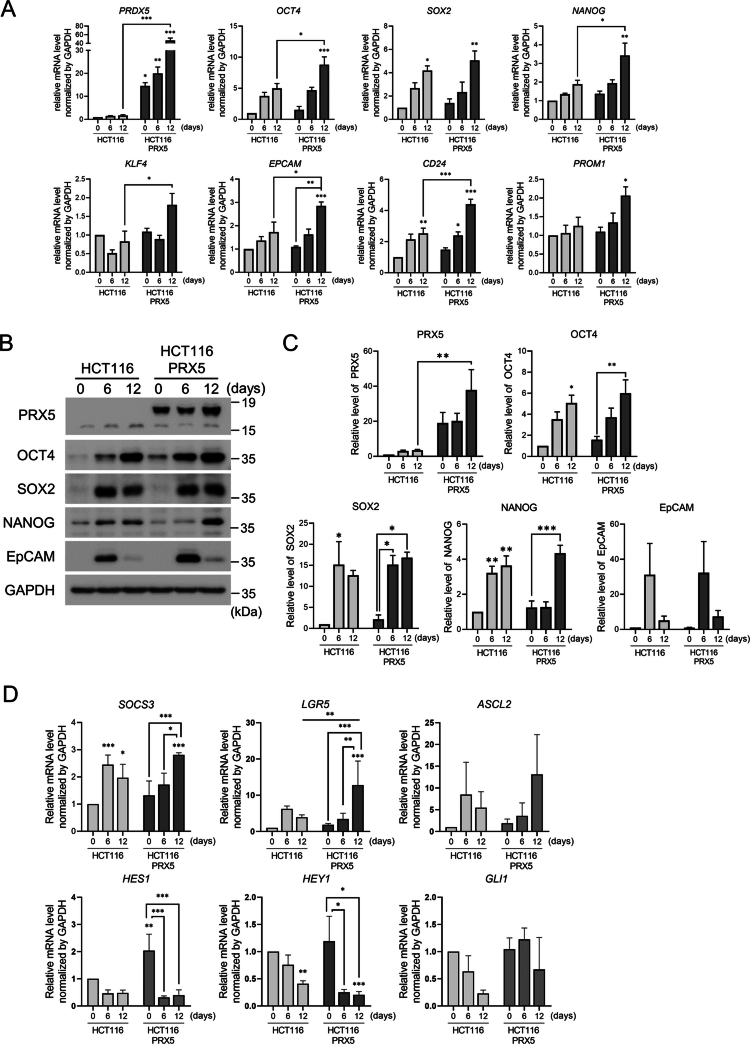
Expression of stemness-associated genes and proteins during spheroid formation in PRX5-overexpressing HCT116 cells. Spheroid samples were collected from HCT116 control and HCT116-PRX5 cells at days 0, 6, and 12 of culturing. (A) Relative mRNA expression levels of *PRX5*, *OCT4*, *SOX2*, *NANOG*, *KLF4*, *CD24*, *CD133*, and EPCAM determined by using qPCR. (B) Western blot analysis of OCT4, SOX2, NANOG, EPCAM, and PRX5 in spheroid lysates collected under the same conditions. (C) Quantification of the protein levels shown in (B). (D) Relative mRNA expression levels of *SOCS3*, *LGR5*, *ASCL2*, *HES1*, *HEY1*, and *GLI1* determined by using qPCR. Data are presented as the mean ± SEM from at least three independent experiments. Statistical significance was determined using two-way ANOVA with Tukey’s post hoc tests (*: *p* < 0.05; **: *p* < 0.01; ***: *p* < 0.001).

To investigate the role of PRX5 in tumorigenicity and spheroid formation, we generated a V5-tagged PRX5 stably expressing the HCT116 cell line (HCT116-PRX5). Successful PRX5 overexpression was confirmed 48 hours post-transfection (Figure S1). We hypothesized that PRX5 drives the CSC phenotype. Over a 12-day induction period, qPCR analysis revealed a time-dependent increase in the core pluripotency factors: OCT4 (1.60-fold), NANOG (1.67-fold), SOX2 (1.25-fold), and KLF4 (1.96-fold) (Figure 4A). Consistent with these findings, EPCAM, CD24, and CD133 were also significantly upregulated 1.61-, 1.97-, and 1.57-fold, respectively (Figure 4A). This transcriptional shift underscores that PRX5 promotes a more aggressive, stem-like state in HCT116 cells.

Western blot analysis confirmed that PRX5 overexpression significantly increased OCT4 (1.23-fold) and SOX2 (1.43-fold) protein levels after 12 days, mirroring the transcriptional data (Figure 4B and C). While NANOG and EPCAM levels remained stable under these conditions, the parallel upregulation of mRNA and protein for OCT4 and SOX2 suggests their direct regulation by PRX5. Functional assays showed that PRX5 overexpression enhanced cellular viability. Specifically, HCT116-PRX5 cells exhibited a 1.26-fold increase in proliferation, as measured by foci formation after 48 h (Figure S2A and S2B). The consistent upregulation of stemness markers combined with enhanced proliferation provides evidence consistent with a role for PRX5 in CSC induction. These data suggest that PRX5 may contribute to the self-renewal program and aggressive tumor growth by stabilizing the stem cell transcriptional signaling.

To further define the signaling context associated with PRX5 overexpression, we analyzed key downstream target genes of major stemness-related pathways by qPCR (Figure 4D). Elevated PRX5 levels were accompanied by increased *SOCS3* expression, which was consistent with enhanced STAT3 activation. In parallel, the Wnt/β-catenin-associated genes *LGR5* and *ASCL2* were upregulated, supporting their involvement in PRX5-driven stemness programs. In contrast, Notch and Hedgehog pathway markers showed no consistent association with PRX5 overexpression. Collectively, these results suggest that PRX5-driven stemness is preferentially supported by STAT3 signaling, with a partial contribution from Wnt/β-catenin-related transcriptional activity. Together, these findings support a model in which PRX5 enhances CSC-associated phenotypes through coordinated regulation of transcriptional and signaling networks.

### 3.5. PRX5 regulates stemness and redox-dependent STAT3 signaling

**Figure 5.**
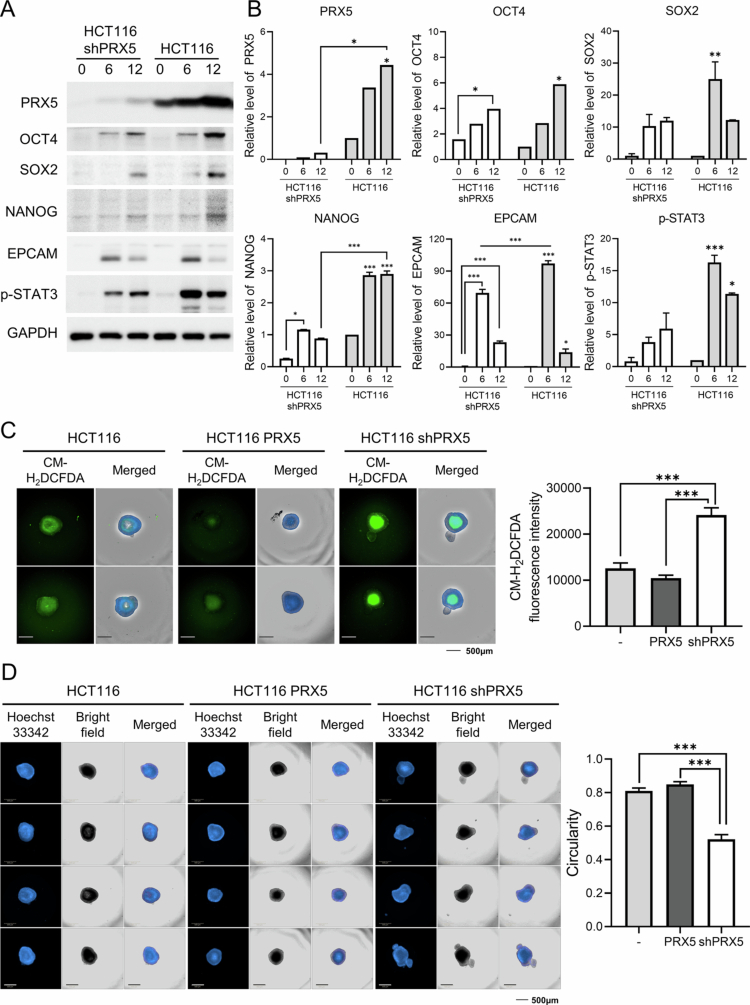
PRX5 regulates stemness-associated factors and intracellular ROS levels. (A) Western blot analysis of PRX, OCT4, SOX2, NANOG, EPCAM, and *p*-STAT3 in spheroid lysates collected under the same conditions. (B) Quantification of the protein levels shown in (A). (C) For ROS measurement and circularity analysis, spheroids from HCT116 WT, PRX5-overexpressing, and PRX5-knockdown cells were cultured for 12 days and stained with CM-H2DCFDA and Hoechst 33242 for 30 min. Images were acquired as z-stacks using the Operetta CLS system in the FITC (exposure time, 400 ms), Hoechst 33242 (15 ms), and bright-field (20 ms) channels. The merged images were generated by combining the FITC, Hoechst 33242, and bright-field channel images, and the mean CM-H2DCFDA fluorescence intensity was quantified. (D) The merged images represent the combined Hoechst 33242 and bright-field channel images, and spheroid circularity was analyzed using ImageJ software. Scale bar = 500 µm. Data are presented as the mean ± SEM from at least three independent experiments. Statistical significance was determined using two-way ANOVA with Tukey’s post hoc tests (*: *p* < 0.05; **: *p* < 0.01; ***: *p* < 0.001).

To understand the functional role of PRX5, we established a stable shPRX5 HCT116 cell line. shPRX5 significantly reduced tumor sphere formation and decreased cancer stem cell frequency, as determined by limiting dilution analysis (Figure S3). Consistently, western blot analysis showed that suppression of PRX5 reduced the expression of stemness-associated markers, including CD133, OCT4, SOX2, NANOG, and EPCAM (Figure 5A and B). In parallel, PRX5 depletion significantly decreased phosphorylated STAT3 (*p*-STAT3) levels, supporting an association between PRX5 expression and STAT3 activation.

To determine whether this regulatory effect was conserved across colorectal cancer cell lines, PRX5 expression was modulated in HT29 and SW480 cells. shPRX5 suppressed CD133, OCT4, NANOG, and EPCAM expression in both cell lines, with SW480 cells showing a particularly reduction in CD133 and EPCAM levels (Figure S4A and S4B). Conversely, PRX5 overexpression increased the expression of CD133, OCT4, and NANOG in HT29 cells, and OCT4 and NANOG in SW480 cells (Figures S4A and S4B). We additionally examined E-cadherin expression as an exploratory marker regarding PRX5-dependent STAT3 signaling. PRX5 suppression resulted in decreased E-cadherin expression in both HT29 and SW480 cells, accompanied by reduced *p*-STAT3 levels (Figure S5A and S5B), suggesting a potential association between PRX5-mediated STAT3 activity and epithelial marker expression.

To further evaluate the role of PRX5 in redox homeostasis and spheroid integrity, intracellular ROS levels and spheroid morphology were analyzed in spheroids derived from HCT116 WT, PRX5-overexpressing, and shPRX5 HCT116 cells after 12 days of culture. CM-H_2_DCFDA staining revealed that PRX5 knockdown markedly increased ROS levels compared with both WT and PRX5-overexpressing spheroids, whereas PRX5 overexpression did not significantly alter ROS levels relative to WT controls (Figure 5C). Consistent with this finding, quantitative analysis of CM-H_2_DCFDA fluorescence intensity showed a significant elevation in the shPRX5 spheroids, indicating that PRX5 depletion disrupted the intracellular redox balance within the spheroids. We next assessed spheroid morphology by measuring circularity. shPRX5 spheroids displayed a lower circularity than both WT and PRX5-overexpressing spheroids, indicating a more irregular and disorganized spheroid structure (Figure 5D). In contrast, PRX5-overexpressing spheroids maintained a compact and rounded morphology comparable to that of WT controls.

**Figure 6.**
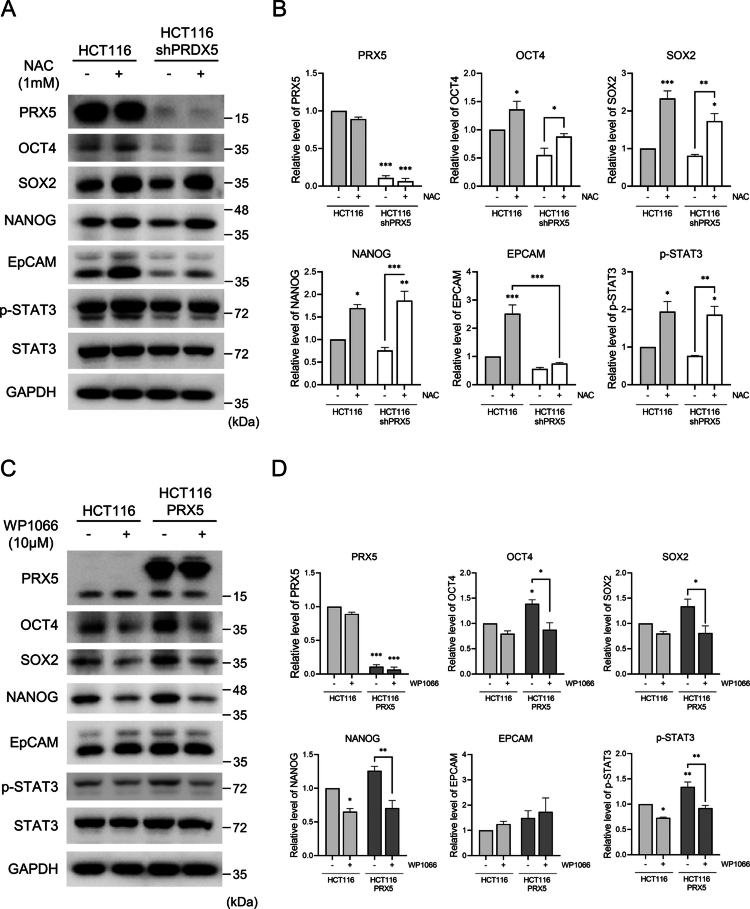
PRX5 regulates STAT3 signaling pathway and its inhibition by NAC and WP1066. (A and B) Spheroids were treated with NAC. (A) Western blot analysis of *p*-STAT3, OCT4, SOX2, NANOG, and EPCAM. (B) Quantification of the protein levels shown in (A). (C and D) Spheroids were treated with the STAT3 inhibitor WP1066. (C) Representative Western blot images. (D) Quantification of the protein levels shown in (C). Data are presented as the mean ± SEM from at least three independent experiments. Statistical significance was determined using one-way ANOVA with Tukey’s post hoc tests (*: *p* < 0.05; **: *p* < 0.01; ***: *p* < 0.001).

To further evaluate whether redox-dependent changes influence STAT3 activation and stemness-associated phenotypes, we performed additional experiments using a ROS scavenger and a STAT3 inhibitor. Treatment with the ROS scavenger *N*-acetylcysteine (NAC) markedly increased *p*-STAT3 levels and significantly upregulated the expression of the core stemness transcription factors OCT4, SOX2, and NANOG in both HCT116 and HCT116 shPRX5 cells (Figure 6A and B). EPCAM expression was strongly increased in HCT116 cells following NAC treatment, whereas only modest changes were observed in PRX5-knockdown cells. Conversely, pharmacological inhibition of STAT3 using WP1066 significantly reduced *p*-STAT3 levels and led to decreased expression of OCT4, SOX2, and NANOG in both HCT116 and PRX5-overexpressing cells (Figure 6C and D). In contrast, EPCAM expression showed minimal or slightly increased changes. Together, these results suggest that PRX5 contributes to the maintenance of redox homeostasis and spheroid structural integrity, particularly through redox-dependent regulation of STAT3 signaling that sustains stemness-associated properties in colorectal cancer cells.

### 3.6. PRX5 overexpression drives cancer stemness and enhanced tumorigenicity via upregulation of pluripotency factors in CRC

**Figure 7.**
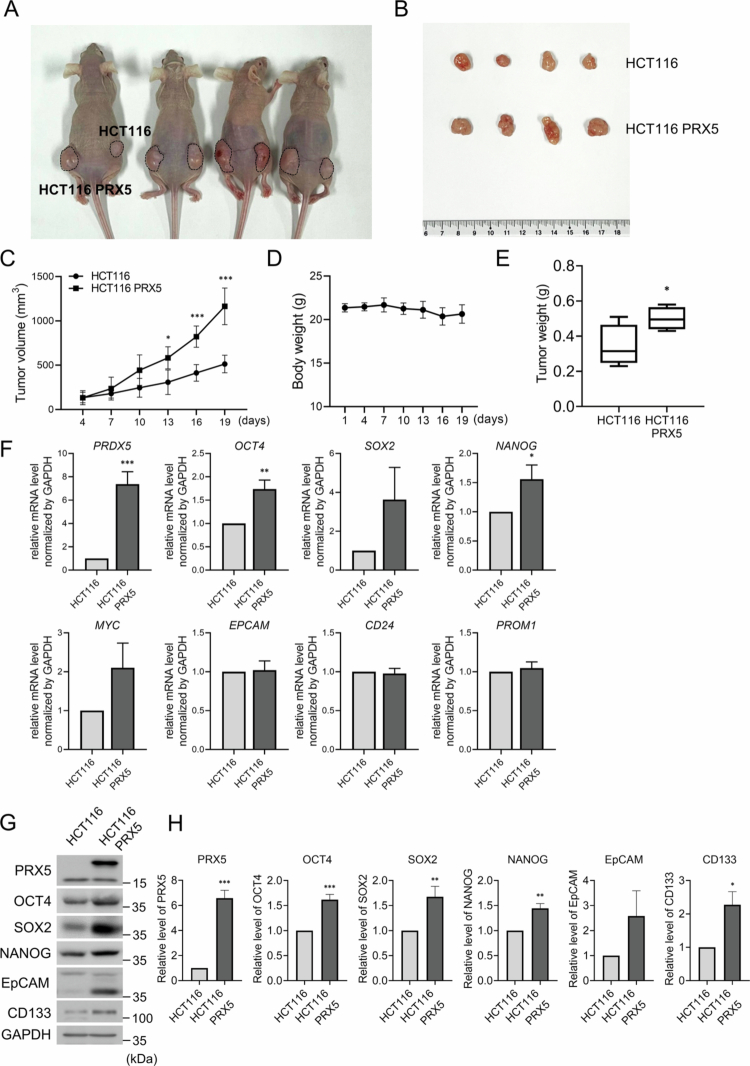
Tumor growth and stemness marker expression in the PRX5-overexpressing HCT116 xenograft model. (A) A representative image of tumor-bearing mice at the experimental endpoint (day 19) following subcutaneous injection of HCT116-control and HCT116-PRX5 cells (5 × 10⁶ cells per flank) into 6-week-old mice. (B) A representative image of excised xenograft tumors collected from the same mice on day 19. (C) Tumor-growth curves showing the mean tumor volume measured every 3 days for both the control and PRX5-overexpressing xenografts. (D) Body weight changes in the mice were monitored throughout the 19-day experimental period. (E) Tumor weights of excised xenografts collected on day 19. (F) Relative mRNA expression levels of PRX5, OCT4, SOX2, NANOG, C-MYC, CD24, CD133, and EPCAM were determined by using qPCR on the xenograft tissues obtained from the control and PRX5-overexpressing groups. (G) Western blot analysis of PRX5, OCT4, SOX2, NANOG, CD133, and EPCAM in xenograft tumor lysates. (H) Quantification of the protein levels shown in (G). The tumor burden did not exceed 5% of the host body weight during the experimental period. Data are presented as the mean ± SEM from at least three independent experiments. Statistical significance was determined using an unpaired two-tailed t-test (*: *p* < 0.05; **: *p* < 0.01; ***: *p* < 0.001).

We used a murine xenograft model to investigate the role of PRX5 in CSC proliferation and tumor progression *in vivo*. We established an HCT116-PRX5 stable cell line and monitored the growth of tumors over 3 weeks after subcutaneously injecting HCT116 WT and HCT116-PRX5 cells into the flanks of nude mice (Figure 7A and B). Remarkably, tumors formed by HCT116-PRX5 grew faster than HCT116 WT tumors, with the difference becoming statistically significant from day 13 post-implantation. The difference in tumor volume was 1.92-fold greater on day 16 and further increased to 2.5-fold on day 19 (Figure 7C). Throughout the three-week observation period, all mice implanted with HCT116 cells maintained stable body weight, indicating that the transplantation procedure and tumor growth did not induce significant systemic stress or adverse side effects (Figure 7D). Following the 3-week observation period, the tumors were excised and weighed. The average weight of HCT116-PRX5 tumors (0.53 g) was approximately 1.82-fold higher than that of HCT116 WT tumors (0.29 g), thereby confirming that the enhanced tumorigenicity mediated by PRX5 overexpression *in vivo* (Figure 7E). We then investigated whether the pro-stemness effects observed in the cell-based assays could be duplicated *in vivo*. To this end, mRNA and protein expression levels were analyzed in tumor tissues harvested 3 weeks post-implantation. Consistent with our *in vitro* data, the mRNA expression levels of OCT4, SOX2, NANOG, and c-MYC were 1.5-fold higher in HCT116-PRX5 tumors compared to the WT group (Figure 7F). Furthermore, PRX5 overexpression in the mouse tissue led to a corresponding 1.25- to 2.2-fold increase in the protein levels of OCT4, SOX2, NANOG, and CD133 and EPCAM than those in the HCT116 WT group (Figure 7G and H).

Collectively, *in vivo* results suggest that PRX5 may function as a contributing factor in promoting the CSC properties of HCT116 cells by upregulating the expression of core stemness-associated genes within the tumor microenvironment, thus leading to PRX5-dependent CSC self-renewal and enhanced tumorigenicity.

## 4. Discussion

The current study suggests PRX5 as a contributing positive regulator of CSC properties and tumorigenicity in HCT116 CRC cells, as indicated by the morphological changes, elevated transcriptional and protein levels, and *in vivo* functional evidence [27–29]. The spheroid culture model successfully induced a CSC subpopulation characterized by the induction of the core pluripotency factors OCT4, SOX2, NANOG, and c-MYC and the functional CSC surface markers CD24, CD44, CD133, and EPCAM, thereby validating the cellular model for subsequent mechanistic analysis [30–32]. We then found that among the PRX family, PRX5 was significantly upregulated during CSC induction, thereby suggesting a specific function for this isoform in HCT116 stemness. Functional studies via siRNA and shRNA-mediated PRX5 knockout and stable PRX5 overexpression led to consistent changes in both CSC marker expression and proliferative capacity, respectively, thereby establishing a consistent association between PRX5 expression and the malignant CSC phenotype. Most importantly, the *in vivo* xenograft experiments provided translational validation, where PRX5 overexpression led to significantly accelerated tumor growth (a 2.5-fold increase in volume) and weight (a 1.82-fold increase), accompanied by marked upregulation of OCT4, SOX2, NANOG, c-MYC, CD133, and EPCAM in the tumor tissue, thus collectively suggesting that PRX5 regulates CRC progression.

While our study suggests that PRX5 contributes to maintaining the expression of core stemness markers and the CSC phenotype in HCT116 cells, the precise molecular regulatory pathways, specifically the intermediary mediators and diverse cell growth signaling cascades associated with PRX5, remain unclear. Nevertheless, PRX5 likely contributes to the maintenance of the CSC phenotype by modulating key signaling factors. First, maintaining a low level of ROS is essential for the self-renewal of CSCs. Wei et al. reported that PRX5 scavenges ROS within mitochondria and peroxisomes, which has been shown to promote the STAT3 phosphorylation [33]. Once activated by PRX5, STAT3 can translocate to the nucleus to upregulate OCT4 and NANOG. Second, Lu and colleagues demonstrated that PRX5 plays a critical role in intracellular redox homeostasis [34]. These findings suggest that PRX5 may stabilize beta-catenin or promote nuclear entry, thereby inducing the expression of CSC markers such as CD44 and CD133. Finally, as PRX5 is localized in the mitochondria, it has been reported to reprogram metabolism in cancer cells to maintain a stem-like state through the regulation of mitochondrial ROS [24]. This metabolic reprogramming may support stemness characteristics through mechanisms that remain to be identified. Furthermore, alternative molecular pathways may support the PRX5-mediated regulation of stemness. Given the functional homology among PRX family members, PRX5 potentially participates in the Hedgehog signaling. In colon cancer cells, PRX2 drives a stem cell-like phenotype via GLI1 with upregulation of OCT4 and NANOG [35]. It is possible that PRX5 could similarly regulate GLI transcription factors to enhance pluripotency gene expression. While PRX5 overexpression has been reported to increase the EMT factor Snail in gastric cancer [36], the direct relevance of this finding to CRC stemness requires further investigation with comprehensive EMT marker panels. Since Snail and Slug are known to activate stemness programs in carcinomas, PRX5-induced Snail could indirectly elevate CSC markers such as OCT4 and NANOG. In antioxidant signaling, PRX5 binds and stabilizes NRF2 in lung cancer cells, and NRF2-driven antioxidant defenses are essential for cancer stem cell self-renewal [37,38]. Thus, PRX5 may enhance CSC traits by enhancing NRF2-dependent gene expression.

To characterize PRX5-specific roles, we first investigated sphere-forming capacity and CSC frequency in a stable shRNA-mediated PRX5 knockout system. Notably, PRX5 depletion significantly reduced CSC frequency, indicating that PRX5 is not merely an incidental byproduct upregulated during CSC induction, but rather a functional regulator essential for sustaining self-renewal potential [39,40]. Moreover, the modulation of PRX5 expression altered the levels of canonical CSC markers, including OCT4, NANOG, EPCAM, and CD133, not only in HCT116 cells but also in HT29 and SW480 cells. This consistent pattern across multiple CRC cell lines suggests that PRX5-dependent regulation of stemness is a conserved mechanism rather than a cell line-specific phenomenon. The marked reduction of CD133 and EPCAM in SW480 further indicates that PRX5 may act as a context-dependent regulator governed by the cellular context.

Our data identify PRX5 as a regulator linking the cellular redox balance to STAT3 signaling in HCT116 colorectal cancer cells. shPRX5 increased intracellular ROS levels and was accompanied by a reduction in STAT3 phosphorylation, whereas PRX5 overexpression lowered ROS and preserved STAT3 activation. These observations support a model in which PRX5 maintains an optimal redox environment that permits STAT3-dependent transcription of key stemness regulators such as OCT4 and NANOG, thereby defining a redox-dependent PRX5-ROS-STAT3 signaling.

STAT3 is a well-established transcriptional regulator in stem cell biology and CSCs. It can directly regulate the expression of pluripotency-related genes, including OCT4 and NANOG, and it also promotes the transcriptional programs required for stemness maintenance [41]. Phosphorylation of STAT3 at Tyr705 is essential for its nuclear translocation and transcriptional activity, and persistent activation of STAT3 (pY705) has been linked to enhanced CSC phenotypes and therapeutic resistance across multiple tumor types [42]. Our results suggest that the reduced *p*-STAT3 levels following shPRX5 regulate the decrease in OCT4, SOX2, and NANOG levels, indicating a functional link between PRX5 and stemness transcription factors. PRX5 localizes to multiple subcellular compartments and functions as an antioxidant enzyme that reduces hydrogen peroxide and other peroxides. Beyond ROS scavenging, PRX5 and related peroxiredoxins have increasingly been recognized as modulators of redox-sensitive signaling pathways. STAT3 activity is sensitive to the intracellular redox state. The moderate ROS can promote STAT3 phosphorylation, whereas excessive oxidative stress can inhibit JAK/STAT signaling. Thus, loss of PRX5 may disrupt redox homeostasis, leading to reduced STAT3 phosphorylation and downstream suppression of stemness-associated transcriptional programs [43]. Notably, our results suggest that PRX5’s role surpasses general antioxidant protection to the regulation of signaling networks that control CSC properties. This is supporting the hypothesis that, despite some functional redundancy among PRX family members, PRX5 can play a role in the CSC niche. The upregulation of PRX5 isoform expression under CSC-inducing conditions in our experiments further supports this finding.

While the present data demonstrate coordinated changes in ROS levels, *p*-STAT3, and stemness marker expression upon PRX5 modulation, the relationship among these components has not yet been formally established through rescue experiments. Nevertheless, multiple independent lines of evidence converge to support the proposed PRX5-ROS-STAT3-stemness regulatory axis. The stable shRNA-mediated PRX5 knockdown elevated intracellular ROS, reduced *p*-STAT3, and suppressed OCT4, SOX2, NANOG, and CD133 expression, while PRX5 overexpression produced reciprocal effects across these same readouts, a pattern more consistent with a linear regulatory pathway than with independent effects. These findings were reproduced in HT29 and SW480 cells, indicating that the regulatory relationship is conserved across CRC models. The proposed model is further supported by established biochemical principles. STAT3 activity is highly sensitive to the intracellular redox environment, with moderate ROS promoting STAT3 phosphorylation through JAK kinase activation and excessive ROS inhibiting signaling through the oxidation of critical cysteine residues in JAK2 and STAT3 [43,44]. Among peroxiredoxin family members, PRX2 has been shown to form a direct redox relay with STAT3 by controlling local H₂O₂ levels [44], and given the broader substrate specificity and multi-compartmental localization of PRX5 [27], analogous regulation is mechanistically conceivable. Downstream, STAT3 directly regulates OCT4 and NANOG transcription by binding to their proximal promoter regions, as demonstrated by ChIP analysis [41,45], providing a defined transcriptional mechanism linking STAT3 activation to the stemness markers examined in this study. However, this model remains to be definitively confirmed through formal rescue experiments. To this end, rescue experiments using constitutively active STAT3 (STAT3-C), the ROS scavenger *N*-acetylcysteine, and STAT3-specific inhibitors are currently underway and will be included in the revised manuscript. Additionally, biochemical interaction studies will be required to determine whether PRX5 regulates STAT3 phosphorylation through direct protein‒protein interactions or indirectly through modulation of the local H₂O₂ concentration that affects upstream JAK kinase activity.

It should be noted that the fold changes observed for individual stemness markers upon PRX5 modulation (1.2–2.0-fold) are modest in their effect size. However, regarding redox signaling, biological responses are governed by concentration-dependent thresholds rather than proportional dose‒response relationships; deviations as small as 2-fold in H₂O₂ levels can shift cellular programs between proliferation, differentiation, and apoptosis [46,47]. The core pluripotency transcription factor network operates within a particularly narrow expression window. Niwa and colleagues demonstrated that less than 2-fold changes in OCT4 expression are sufficient to redirect embryonic stem cell fate decisions, establishing that small quantitative changes produce qualitative phenotypic switches [48]. STAT3 transcriptional output is similarly sensitive to changes in phosphorylation levels due to cooperative DNA-binding dynamics at target promoters [49]. Published studies on CSC regulators in CRC, including PRX2, have reported biologically meaningful effects within a comparable 1.3–1.8-fold range that are correlated with significant functional outcomes [34,35]. Critically, the fold changes in our study are consistently reproduced across multiple molecular levels, multiple independent markers (OCT4, SOX2, NANOG, CD133, EPCAM), bidirectional perturbation strategies, multiple functional readouts, and HCT116, HT29, and SW480 cells. Importantly, in vitro molecular changes were amplified to a 2.5-fold increase in tumor volume and a 1.82-fold increase in tumor weight in PRX5-overexpressing xenografts (Figure 7), which is a hallmark of regulatory nodes functioning at critical pathway control points. These considerations support the conclusion that PRX5 functions as a biologically meaningful, though not sole, regulator of the CSC phenotype in CRC. In addition to the redox-dependent STAT3 signaling axis described here, emerging evidence suggests that PRX5 may participate in ferroptosis-associated pathways, a process closely linked to ROS-dependent lipid peroxidation [50,51]. In line with this, PRX5 has also been proposed as a potential therapeutic target in multiple cancer contexts, including colorectal and castration-resistant prostate cancers [52].

We note that mRNA and protein levels did not uniformly correlate for all markers examined, a discrepancy that reflects biological mechanisms rather than experimental issues. In particular, NANOG showed significant mRNA upregulation that was not consistently reflected at the protein level, likely due to its extensive post-transcriptional regulation through AU-rich element-mediated mRNA destabilization, ubiquitin-dependent proteasomal degradation via E3 ligases such as FBXW8, and redox-sensitive protein stability [53,54]. Given that PRX5 modulation alters intracellular ROS levels, it is plausible that redox-dependent changes in NANOG protein stability partially antagonize the transcriptional upregulation, creating a compensatory mechanism. Similarly, EPCAM protein levels were more prominently affected in vivo than in vitro, which is consistent with the known regulation of EPCAM surface expression by regulated intramembrane proteolysis via TACE/ADAM17 and *γ*-secretase, post-translational glycosylation, and microenvironmental signals that are more fully represented in the xenograft model [55,56]. Such an mRNA-protein gap is a well-known genome-wide phenomenon, with correlations of only *r* = 0.4–0.6 between transcript and protein abundance [57]. Importantly, for those markers where mRNA-protein correlation was observed (OCT4, SOX2), the consistent changes across both molecular levels promote the validity of the regulatory effects identified.

Although E-cadherin expression was reduced upon PRX5 knockdown in HT29 and SW480 cells (Figure S5A and S5B), this single-marker observation is insufficient to conclude that PRX5 directly regulates epithelial‒mesenchymal transition. A comprehensive assessment would require analysis of a full panel of epithelial markers and mesenchymal, together with functional migration and invasion assays. The reduced E-cadherin upon PRX5 depletion may reflect disruption of epithelial integrity or changes in STAT3-dependent E-cadherin transcription rather than activation of EMT program. Notably, STAT3 has been reported to regulate E-cadherin expression in cellular environments, suggesting that the observed E-cadherin reduction may be a secondary consequence of decreased *p*-STAT3 levels rather than an independent EMT event. Recent studies have shown that CSCs frequently reside in hybrid epithelial/mesenchymal (E/M) states that do not conform to the canonical binary EMT framework [58,59]. The relationship between PRX5, redox homeostasis, and epithelial flexibility requires dedicated investigation in future studies employing comprehensive marker and functional assays.

A limitation of the present study is that it does not definitively establish a relationship between PRX5 loss, a reduction of *p*-STAT3, and decreased stemness marker expression. Future studies should include functional rescue experiments using constitutively active STAT3 to determine whether the restoration of STAT3 activity can recover OCT4/NANOG expression and CSC phenotypes in PRX5-deficient cells. To further strengthen our conclusions, further studies are needed. These include pharmacological modulation of the JAK/STAT pathway and ChIP assays to confirm *p*-STAT3 binding at the OCT4 and NANOG promoters. Additionally, time-course experiments would help clarify the temporal sequence of changes in ROS levels, *p*-STAT3 activation, and stemness marker expression [23,41].

A further limitation is that the in vivo tumor-initiating cell frequency was not directly quantified by limiting dilution transplantation (LDT). Although in vitro ELDA demonstrated that PRX5 depletion significantly reduced CSC frequency, and single-dose xenograft experiments showed enhanced tumor growth (2.5-fold greater volume, 1.82-fold higher weight) and elevated stemness markers in PRX5-overexpressing tumors, formal in vivo limiting dilution assays represent the gold standard for quantifying tumor-initiating cell frequency. By injecting cells into both the left and right sides of each mouse, we compared the groups within the same animal. This approach ensures that the observed growth differences are not due to individual variations between the mice. Nevertheless, future studies should incorporate in vivo LDT with serial passage to definitively establish that PRX5 modulates the tumor-initiating cell frequency and that PRX5-dependent CSC enrichment is maintained across serial transplantation, which represents the gold standard for demonstrating self-renewal capacity [60,61]. Such experiments would enable precise quantification of the minimum cell number required for tumor initiation and assessment of whether PRX5 overexpression reduces this threshold, thereby providing definitive evidence for enhanced tumor-initiating capacity rather than enhanced proliferative capacity alone.

In summary, our findings propose a model in which PRX5 maintains a permissive redox environment that enables STAT3 activation and the transcriptional maintenance of CSC-associated programs in colorectal cancer cells. These results suggest that PRX5 as a potential molecular target for strategies to disrupt CSC maintenance through redox-based approaches. Further mechanistic and *in vivo* validation will be required to determine the therapeutic possibility of targeting the PRX5-ROS-STAT3 signaling.

## Supplementary Material

Supplementary Data.docx Supplementary Data.docx

ARRIVE guidelines Author Checklist 28th_Apr_26.pdf ARRIVE guidelines Author Checklist 28th_Apr_26.pdf

## Data Availability

Any additional information to support the findings in the study is available from the corresponding authors on reasonable request https://figshare.com/s/3bb7b079edbb8ade7993.

## Supplemental material

Supplemental data for this article can be accessed online at https://figshare.com/s/3bb7b079edbb8ade7993.
