# Supplementary material for: PRX5 as a critical driver of colorectal cancer stemness and tumorigenicity
Source: Redox Rep. 2026 May 16;31(1):2673661. doi: 10.1080/13510002.2026.2673661 (PMC13182158; doi:10.1080/13510002.2026.2673661)
Supplement: Supplementary Data.docx [file YRER_A_2673661_SM5386.docx]

**Supplementary Data**

**Supplementary methods**

**Cell culture and spheroid formation**

HT29 and SW480 human colon cancer cells were purchased from the American Type Culture Collection. HT29 cells were maintained in DMEM and SW480 cells were maintained in RPMI-1640 (Welgene) at 37 °C in a humidified incubator with 5% CO_2_. Both media were supplemented with 10% FBS, 100 U/mL penicillin, and 100 μg/mL streptomycin. Cells was detached with trypsin to prepare a single-cell suspension, which was seeded into ultra-low attachment 96-well plates at a density of 1x10^3^ cells/well. Spheroids were maintained for up to 12 days in DMEM/F-12 medium supplemented with 20 ng/mL EGF, 10 ng/mL bFGF, 5 μg/mL insulin, and 1x B-27 supplement.

**Plasmid and lentivirus generation**

Two independent PRX5-targeting short hairpin RNA (shRNA) constructs in the pLKO.1 backbone (shPRX5#1 and shPRX5#2) were obtained from Sigma-Aldrich. HEK293FT cells were cultured until 70–80% confluence and transfected with shRNA plasmid, packaging vector psPAX2, and enveloping vector pMD.2G using the Effectene transfection reagent (Qiagen). 12 hours after transfection, the medium was replaced with fresh medium, and after a further 24-48 hours, the lentivirus-containing medium was harvested and purified using a 0.45-µm filter. Where indicated, viral supernatants were concentrated using an Amicon® Ultra-15 centrifugal filter unit (Millipore).

**Stable cell line**

To generate stable PRX5-overexpressing and PRX5-knockdown HT29 and SW480 cell lines, cells were infected with lentiviruses encoding PRX5 (pLenti6.3-PRX5) or two independent PRX5-targeting shRNAs (pLKO.1-shPRX5#1 and pLKO.1-shPRX5#2) in the presence of 8 μg/mL polybrene. After 48 hours, PRX5-overexpressing cells were selected with 8 μg/mL blasticidin (Sigma-Aldrich), whereas shRNA-transduced cells were selected with 1 μg/mL puromycin (Sigma-Aldrich) for 1 week. The resulting stable cell lines were designated HT29 PRX5, SW480 PRX5, HT29 shPRX5#1/#2, and SW480 shPRX5#1/#2 and were expanded for subsequent experiments.

**Statistical analysis**

Data are presented as the mean ± standard error of the mean (SEM) of three independent experiments. Statistically significant differences were determined by using unpaired two-tailed Student’s t-tests, one-way ANOVA using the GraphPad Prism 8 software (GraphPad Software, San Diego, CA, USA). After one-way ANOVA, Tukey’s post hoc tests were conducted for intergroup comparisons. Results with p < 0.05 were considered statistically significant. Asterisks indicate calculated p-values as follows: *: p < 0.05; **: p < 0.01; ***: p < 0.001.

**Supplementary figures**


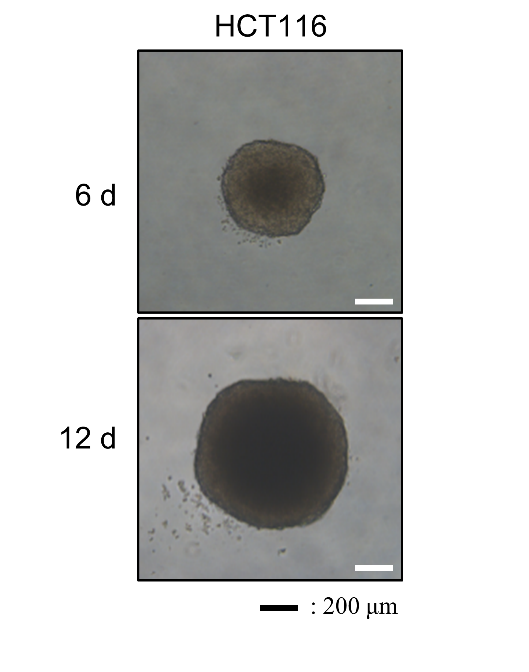


**Fig. S1. Induction and characterization of a CSC-like subpopulation in HCT116 cells.** Representative live-cell images of spheroids at day 6 and day 12. Scale bar = 200 um.


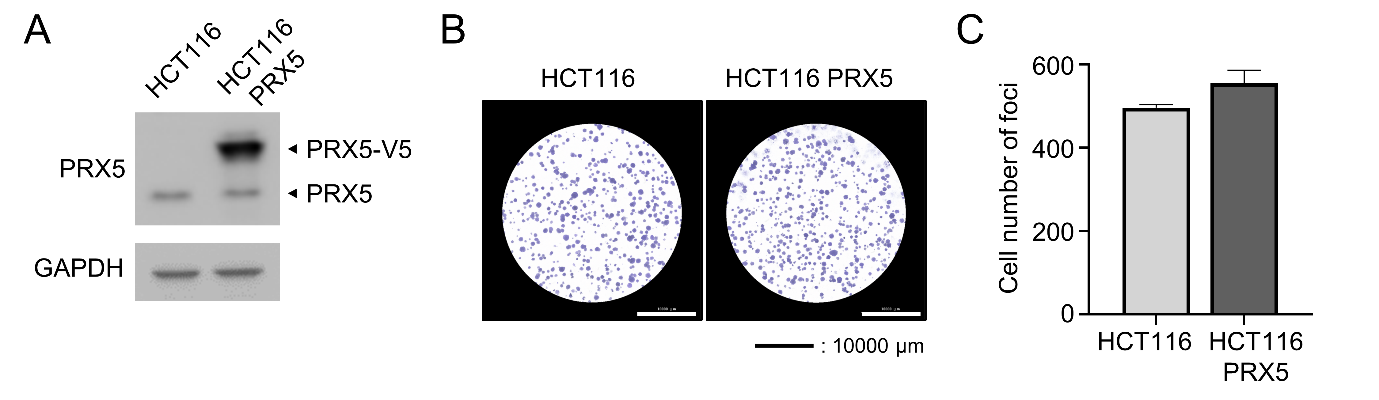


**Fig. S2. PRX5 overexpression potentiates stemness features and proliferative capacity in HCT116 cells.** (A) Western blot analysis confirming stable expression of V5-tagged PRX5 in HCT116-PRX5 cells, showing a characteristic double-band pattern corresponding to the tagged construct. (B) Representative images of clonogenic assays comparing colony formation between HCT116 control and PRX5-overexpressing cells. Scale bar = 10000 um. (C) Quantification of colonies larger than 300 μm in diameter based on images shown in (B). Data are presented as the mean ± SEM from at least three independent experiments. Statistical significance was determined using two-way ANOVA with Tukey’s post hoc tests (*: p < 0.05).


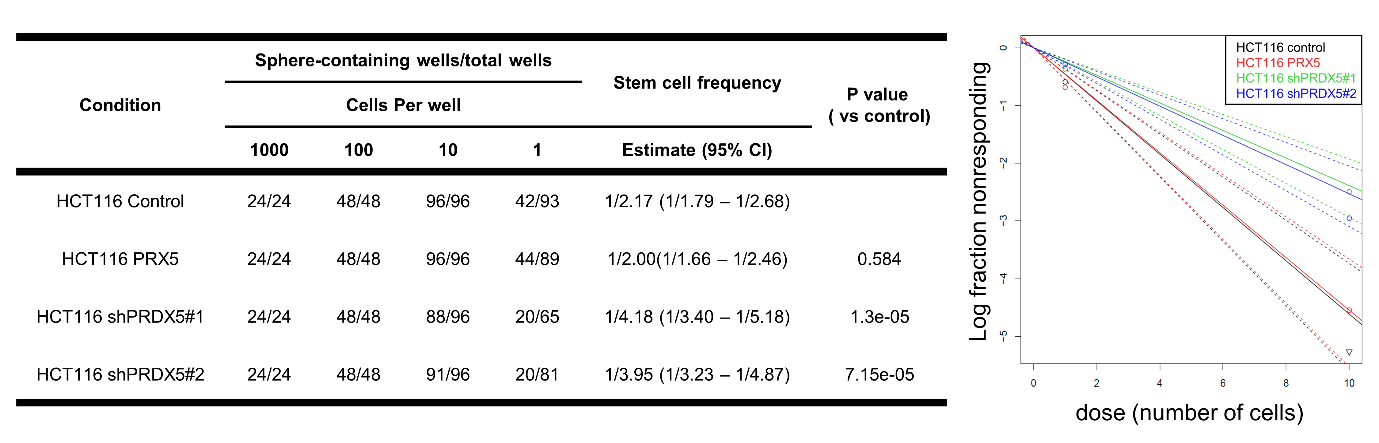


**Fig. S3. PRX5 depletion reduces stem cell frequency in HCT116 cells as assessed by ELDA.** (A) In vitro ELDA of HCT116 cells. Estimated stem cell frequency for each group, expressed as 1/(stem cell frequency) with 95% confidence intervals. Limiting dilution data plotted as log fraction of non-responding wells versus cell dose. Points represent observed values, and lines indicate model-derived fits based on the single-hit assumption.


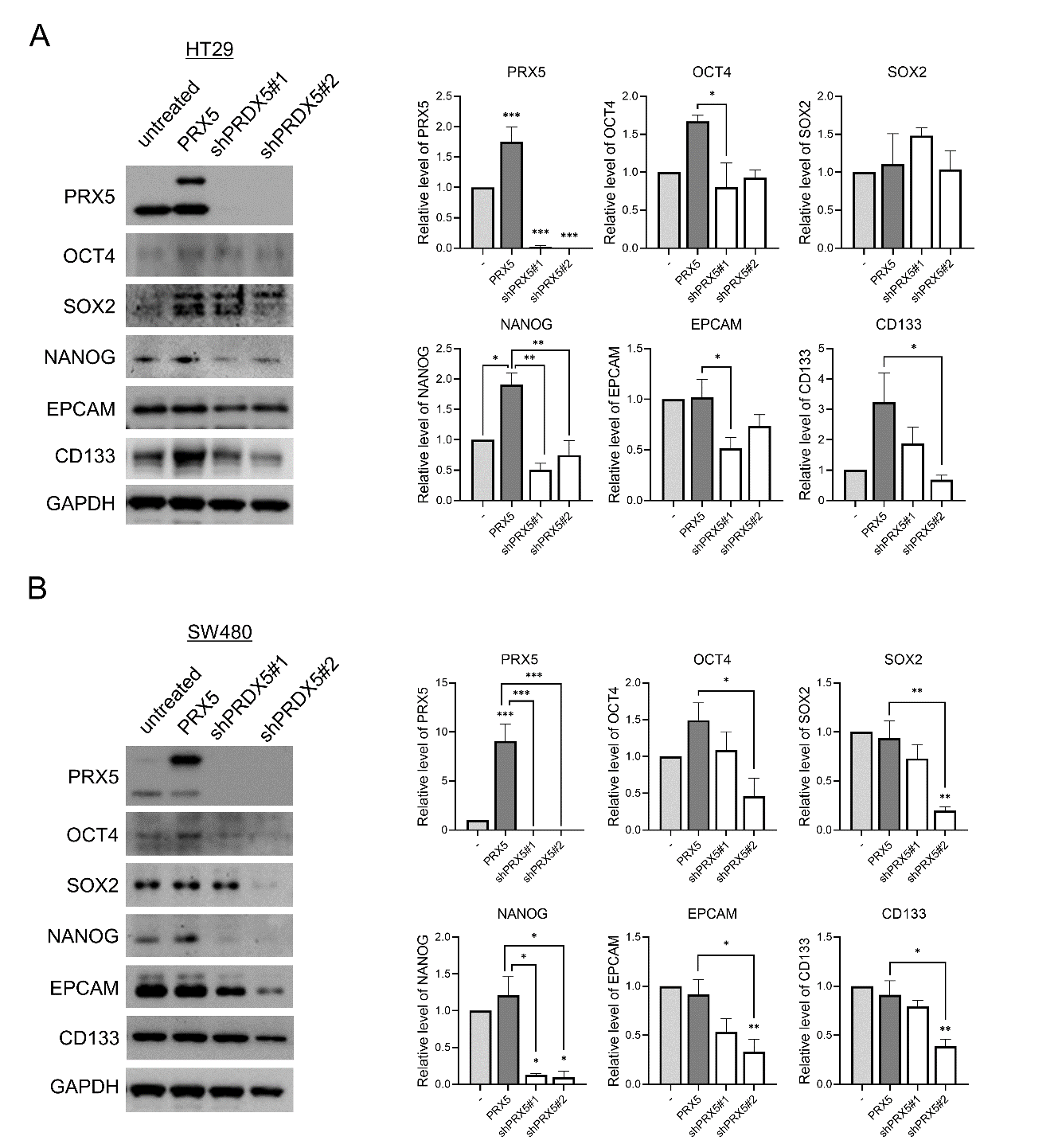


**Fig. S4. Expression of stemness-associated markers in HT29 and SW480 spheroids under PRX5 modulation.** HT29 (control, PRX5-overexpressing, shPRDX5#1, and shPRDX5#2) and SW480 (control, PRX5-overexpressing, shPRDX5#1, and shPRDX5#2) cells were cultured as spheroids in serum-free condition for 12 days. (A) Representative Western blot images and quantification of stemness-associated proteins (PRX5, OCT4, SOX2, NANOG, EPCAM, and CD133) in HT29 spheroids. (B) Representative Western blot images and quantification of the indicated proteins in SW480 spheroids. Data are presented as the mean ± SEM from at least three independent experiments. Statistical significance was determined using one-way ANOVA with Tukey’s post hoc tests (*: p < 0.05; **: p < 0.01; ***: p < 0.001).


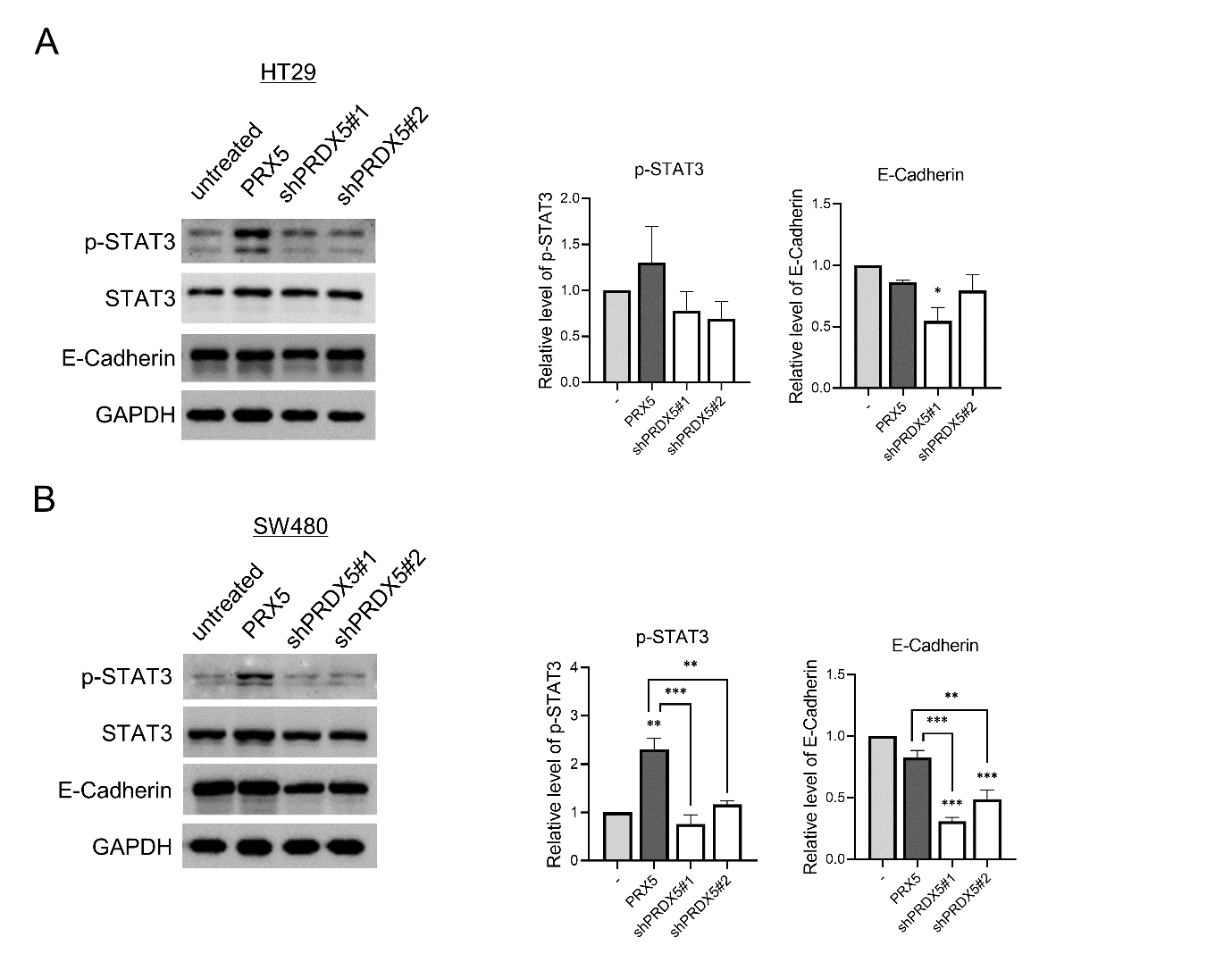


**Fig. S5. Expression of p-STAT3 and EMT marker in HT29 and SW480 spheroids under PRX5 modulation.** HT29 (control, PRX5-overexpressing, shPRDX5#1, and shPRDX5#2) and SW480 (control, PRX5-overexpressing, shPRDX5#1, and shPRDX5#2) cells were cultured as spheroids in serum-free condition for 12 days. (A) Representative Western blot images and quantification of p-STAT3 and epithelial marker E-Cadherin in HT29 spheroids. (B) Representative Western blot images and quantification of the indicated proteins in SW480 spheroids. Data are presented as the mean ± SEM from at least three independent experiments. Statistical significance was de-termined using one-way ANOVA with Tukey’s post hoc tests (*: p < 0.05; **: p < 0.01; ***: p < 0.001).

**Supplementary Table 1.** List of primer sequences

| **Gene** | **Primer sequence** | |
| --- | --- | --- |
|  | **Forward (5' - 3')** | **Reverse (5' - 3')** |
| *OCT4* | ACATCAAAGCTCTGCAGAAA | CTGAATACCTTCCCAAATA |
| *SOX2* | CGGAAAACCAAGACGCTCAT | TGTGCGCGTAACTGTCCAT |
| *NANOG* | ATGGTGTGACGCAGGGATG | GGACTGGATGTTCTGGGTCTG |
| *C-MYC* | GCCTTGGTTCATCTGGGTCT | TGCTTAGGAGTGCTTGGGAC |
| *EPCAM* | TGATCCTGACTGCGATGAGAG | CTTGTCTGTTCTTCTGACCCC |
| *CD24* | GCACTGCTCCTACCCACG | GCAGAAGAGAGAGTGAGACCAC |
| *CD44* | TCTGTGCAGCAAACAACACA | TAGGGTTGCTGGGGTAGATG |
| *CD133* | AGCTACTTGGCTCAGACTGG | CCTCAAATTTCCTGGGGGCA |
| *KLF4* | GATGATGCTCACCCCACCTT | TGTGCCTTGAGATGGGAACTC |
| *PRDX1* | CCACGGAGATCATTGCTTTCA | AGGTGTATTGACCCATGCTAGAT |
| *PRDX2* | CCTTCCAGTACACAGACGAGCA | CTCACTATCCGTTAGCCAGCCT |
| *PRDX3* | GAAGTTGTCGCAGTCTCAGTGG | CACCGTAGTCTCGGGAAATCTG |
| *PRDX4* | CGCTTTTGGCGACAGACTTGAAG | CCAAGTCCTCCTTGTCTTCGAG |
| *PRDX5* | CTGGAGCTCTGAAGGCCAAG | CCAGGAGCCGAACCTTGC |
| *PRDX6* | CAGCTACCACTGGCAGGAACTT | GGAAGGACCATCACACTATCCC |
| *SOCS3* | CATCTCTGTCGGAAGACCGTCA | GCATCGTACTGGTCCAGGAACT |
| *LGR5* | CCTGCTTGACTTTGAGGAAGACC | CCAGCCATCAAGCAGGTGTTCA |
| *ASCL2* | CGTGAAGCTGGTGAACTTGG | GGATGTACTCCACGGCTGAG |
| *HES1* | TCAACACGACACCGGATAAA | CCGCGAGCTATCTTTCTTCA |
| *HEY1* | TGTCTGAGCTGAGAAGGCTGGT | TTCAGGTGATCCACGGTCATCTG |
| *GLI1* | AGCCTTCAGCAATGCCAGTGAC | GTCAGGACCATGCACTGTCTTG |
| *GAPDH* | GGAAGGACTCATGACCACAGT | GGATGATGTTCTGGAGAGCCC |

**Supplementary Table 2.** List of materials

| **Antibodies** | | |
| --- | --- | --- |
| **Products** | **Product number** | **Manufacturer** |
| GAPDH (G-9) | sc-365062 | Santa Cruz |
| Oct-4 Antibody | 2750 | Cell Signaling Technology |
| Sox2 (D6D9) Rabbit Monoclonal Antibody | 23064 | Cell Signaling Technology |
| Nanog (D73G4) Rabbit Monoclonal Antibody | 4903 | Cell Signaling Technology |
| EpCAM (E6V8Y) Rabbit Monoclonal Antibody | 93790 | Cell Signaling Technology |
| CD133 (D2V8Q) Rabbit Monoclonal Antibody | 64326 | Cell Signaling Technology |
| Phospho-Stat3 (Tyr705) (D3A7) Rabbit  Monoclonal Antibody | 9145 | Cell Signaling Technology |
| Stat3 (79D7) Rabbit Monoclonal Antibody | 4904 | Cell Signaling Technology |
| BD Transduction Laboratories™ Purified  Mouse Anti-E-Cadherin | 610181 | BD Biosciences |
| Peroxiredoxin 1 | LF-PA50608 | Abfrontier |
| Peroxiredoxin 2 | 10545-2-AP | Proteintech |
| Peroxiredoxin 3 (AF17D2FF) | LF-MA0329 | Abfrontier |
| Peroxiredoxin 4 | LF-PA0009 | Abfrontier |
| Peroxiredoxin 5 | LF-PA0210 | Abfrontier |
| Peroxiredoxin 6 (1A11) | LF-MA0013 | Abfrontier |
| V5 Tag (SV5-Pk1) | E12-005 | Enogene |
| Anti-rabbit IgG, HRP-linked Antibody | 7074 | Cell Signaling Technology |
| Goat anti-Mouse IgG Fc Secondary Antibody,  HRP | 31437 | Invitrogen |
| **Chemicals, recombinant proteins and reagents** | | |
| **Products** | **Product number** | **Manufacturer** |
| Human EGF, Animal-Free Recombinant Protein | AF-100-15 | Peprotech |
| Recombinant Human FGF-basic (154 a.a.) | 100-18B | Peprotech |
| insulin from bovine pancreas | I6634 | Sigma aldrich |
| Lipofectamine RNAiMAX | 13778 | Thermo Fisher Scientific |
| DMEM | LM001-05 | Welgene |
| Fetal Bovine Serum | 16000-044 | Gibco |
| Penicillin-Streptomycin(100X) | LS202-02 | Welgene |
| Trypsin-EDTA (1X) | LS015-10 | Welgene |
| DMEM / F-12 1:1 Mixture(1X) | LM002-04 | Welgene |
| PrimeSTAR® GXL DNA Polymerase | R050A | TaKaRa |
| In-Fusion® Snap Assembly | 638948 | Clontech |
| Effectene Transfection Reagent | 301425 | QIAGEN |
| Minisart® Syringe Filter,  Polyethersulfone (PES), Pore Size 0.45 µm | 16537-K | Satorius |
| Blasticidine S hydrochloride | 15205-25MG | Sigma aldrich |
| PRO-PREP™ Protein Extraction Solution (C/T) | 17081 | iNtRON |
| Halt™ Protease and Phosphatase Inhibitor Cocktail (100X) | 78440 | Thermo Fisher Scientific |
| 40% Acrylamide/Bis Solution, 29:1 (Bradford assay) | 1610146 | Bio-Rad |
| Amersham™ Protran® Western blotting membranes, nitrocellulose | 10600001 | Cytiva |
| Skim milk | 232100 | BD Difco |
| CM-H2DCFDA | C6827 | Thermo Fisher Scientific |
| Hoechst 33342 | H1399 | Thermo Fisher Scientific |
| N-acetyl-L-cysteine | A7250 | Sigma aldrich |
| STAT3 inhibitor III, WP 1066 | 573097 | Sigma aldrich |
| 2,2,2-tribromoethanol | 48402 | Sigma aldrich |
